# Pathogenesis of Spontaneous and Experimental Appendicitis, Ulcer of the Stomach and Cholecystitis

**Published:** 1916-04

**Authors:** 


**Pathogenesis of Spontaneous and Experimental Appendicitis, Ulcer of the Stomach and Cholecystitis,** Edward C. Rosenow, M. D., Rochester, Minn. Jour. Ind. State Med. Assn., October, 1915. Elective Localization of Streptococci following intravenous injection from Appendicitis, Ulcer of the Stomach and Cholecystitis.

| Source of Streptococci | Time of use | Strain | Animals Injected | Percentage of Animals Showing Lesions in: Appendix | Stom. Hem. | Duod. Ul. | Gallbladder | Pancreas | Intestines |
|---|---|---|---|---|---|---|---|---|---|
| Appendicitis— | When isolated | 14 | 68 | 68 | 6 | 1 | 1 | 0 | 9 |
| | Later | 8 | 26 | 15 | 19 | 15 | 4 | 0 | 0 |
| | After animal passage | 7 | 22 | 45 | 45 | 30 | 40 | 0 | 20 |
| Ulcer of stomach in man— | | | | | | | | | |
| | When isolated | 18 | 103 | 2 | 60 | 60 | 20 | 3 | 7 |
| | Later | 8 | 22 | 5 | 5 | 0 | 5 | 0 | 0 |
| | After animal passage | 7 | 39 | 0 | 23 | 33 | 30 | 15 | 15 |
| Cholecystitis— | | | | | | | | | |
| | When isolated | 12 | 41 | 0 | 29 | 15 | 80 | 5 | 17 |
| | Later | 5 | 14 | 14 | 28 | 14 | 7 | 0 | 0 |
| | After animal passage | 4 | 16 | 0 | 31 | 13 | 56 | 19 | 13 |
| Average per cent. of animals showing lesions in these organs exclusive of specific strains | | | | 5 | 20 | 9 | 11 | 6 | 8 |

